# Facial Natural Killer/T-Cell Lymphoma: A Deadly Sinusitis Mimic

**DOI:** 10.7759/cureus.22095

**Published:** 2022-02-10

**Authors:** Rachel E Bridwell, Steve C Gillis, Gerrit W Davis, Brannon L Inman, Brit Long

**Affiliations:** 1 Emergency Medicine, Madigan Army Medical Center, Joint Base Lewis-McChord, USA; 2 Flight Paramedic, Army Special Operations Aviation Command, Fayetteville, USA; 3 Emergency Medicine, Brooke Army Medical Center, Fort Sam Houston, USA

**Keywords:** secondary hemophagocytic lymphohistiocytosis (hlh), extra-nodal, lymphoma, facial, t-cell, nk cell, facial swelling

## Abstract

Sinusitis and pre-septal cellulitis are common emergency department (ED) conditions, though rare and lethal mimics can present in a similar manner. We present a case of natural killer (NK)/T-cell lymphoma mimicking sinusitis and pre-septal cellulitis. Diagnosis of this condition may include imaging modalities such as CT and MRI, though definitive diagnosis requires tissue biopsy. Therapeutic interventions involve chemotherapy and radiation, with little role for surgical debridement. Complications in treatment can occur including hemophagocytic lymphohistiocytosis. Despite standard treatments, mortality remains high for cases of facial lymphoma.

## Introduction

Sinusitis and pre-septal cellulitis are common emergency department (ED) conditions, affecting 31 million adults annually and accounting for one in five of all adult antibiotic prescriptions [1-2]. While emergency physicians see a myriad of sinusitis and pre-septal cellulitis, other conditions may mimic this presentation. One such nefarious etiology includes natural killer (NK)/T-cell lymphoma (NKTL), an extranodal Non-Hodgkin’s Lymphoma, which can rarely present as isolated facial swelling [3-5]. However, it more commonly presents with nasal complaints such as rhinorrhea, epistaxis, or chronic congestion [3-5]. We present a case of a young female with subacute facial and periorbital edema with cranial nerve deficits resembling sinusitis and pre-septal cellulitis refractory to appropriate antibiotics with an eventual diagnosis of primary NKTL.

## Case presentation

A 19-year-old woman was transferred to the emergency department (ED) from an outlying urgent care clinic with complaints of progressively worsening left-sided facial numbness, swelling, and left eye pain with movement over one month. Ten days prior to her acute presentation, she underwent a left maxillary antrostomy. Additional surgical history included left frontal sinusotomy, left endoscopic total ethmoidectomy, and left endoscopic maxillary antrostomy 12 months prior due to Pott’s puffy tumor. She denied facial trauma, visual changes, blurry vision, diplopia, epistaxis, fever, chills, nausea, or vomiting.

**Figure 1:**
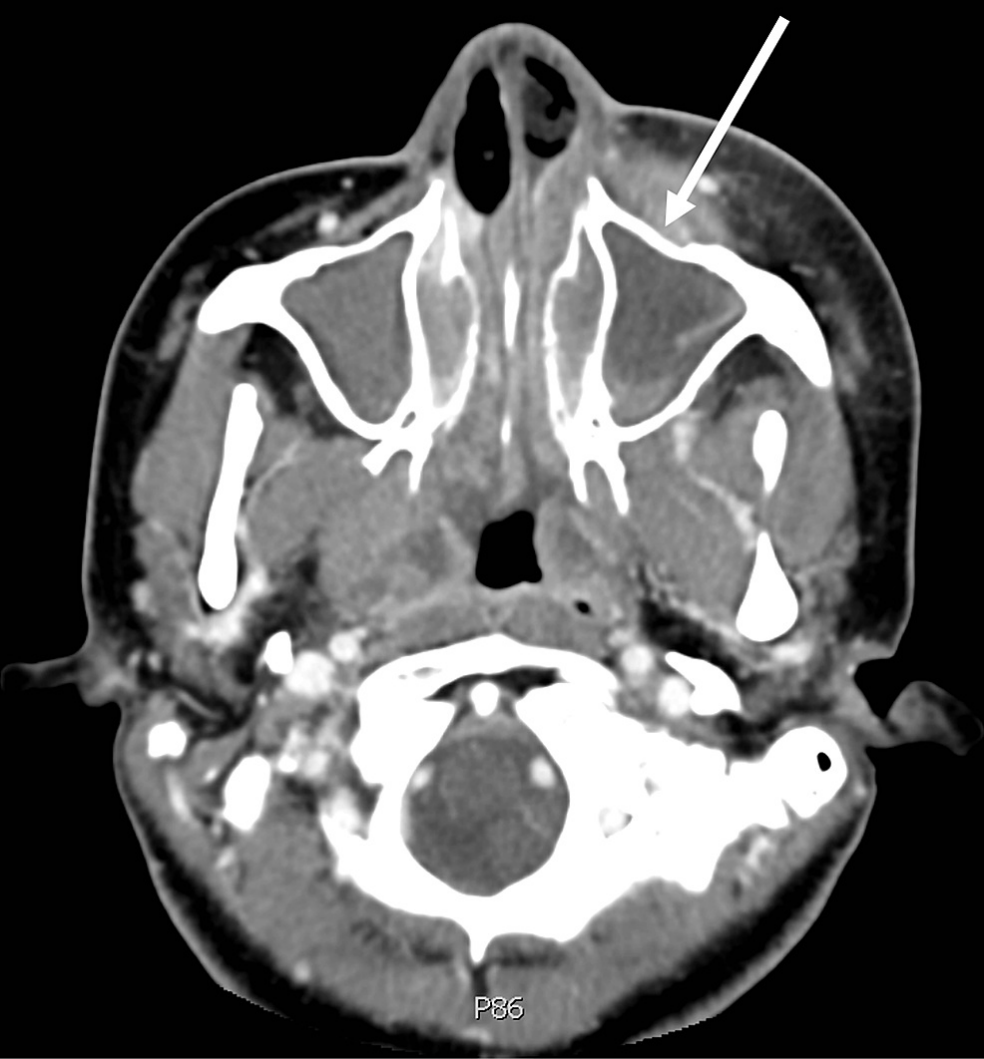
Axial slice of a CT with contrast of the sinuses demonstrating complete opacification of parasinuses and nasal cavity with aggressive osseous changes (white arrow) and diffuse fat stranding with concern for invasive sinusitis with pre- and post-septal orbital tissue involvement.

**Figure 2:**
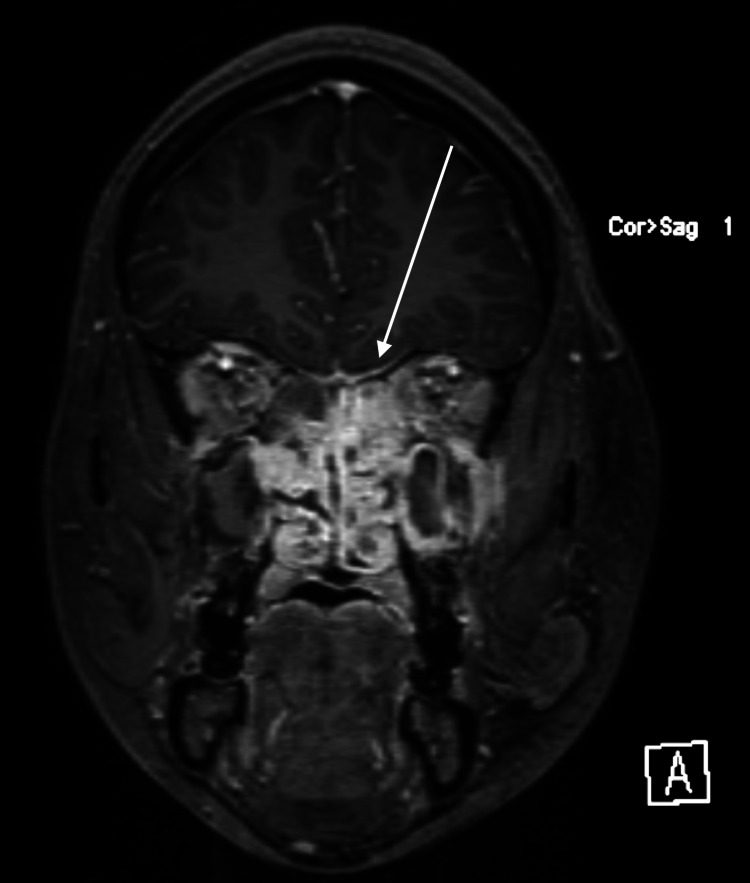
MRI with contrast of the head and maxillofacial area demonstrating inflammation of the left maxillary and sphenoid sinus cavities as well as orbital involvement. Additional linear dural enhancement along the anterior cranial fossa floor without brain parenchyma involvement demonstrates early epidural involvement (white arrow).

The patient’s initial vital signs were blood pressure 109/76 mmHg, heart rate 78 beats per minute, respiratory rate 16 breaths per minute, and oxygen saturation 99% on room air with a temperature of 98.3 degrees Fahrenheit. Physical examination was notable for significant erythema, edema, and tenderness of the left facial and periorbital area, preventing eye opening. Her pupils were equal, round, and reactive to light. Her extraocular movements were intact but painful. Cranial nerve examination demonstrated deficits including left facial weakness, left numbness in the V2 distribution, left hearing deficit, dysphagia, and a new absence of gag reflex. A complete blood count demonstrated a lymphopenia of 1,930/mm^3, and the basic metabolic panel was remarkable only for hyponatremia of 132 mEq/L. A contrasted CT demonstrated complete opacification of the parasinuses and nasal cavity with aggressive osseous changes and diffuse fat stranding, concerning invasive sinusitis with pre- and post-septal orbital tissue involvement (Figure 1). Subsequent MRI showed inflammation of the left maxillary and sphenoid sinus cavities as well as orbital involvement and dural enhancement signifying leptomeningeal involvement (Figure 2). Due to the clinical presentation and lymphopenia, vancomycin, ceftriaxone, metronidazole, and voriconazole were initiated to target invasive bacterial and fungal infections. Otolaryngology, ophthalmology, and infectious disease were consulted, and the patient was taken for operative management.

The patient underwent serial debridements with otolaryngology, as pathology and surgical findings were inconsistent with fungal infection. Wound cultures grew *Staphyloccocus aureus* and *Streptococcus pyogenes*, but the inflammation did not improve as anticipated with surgical intervention and antibiotics. After tissue immunostaining, the patient was diagnosed with localized NKTL lymphoma. The patient was transitioned to a modified steroid-methotrexate-ifosfamide-L-asparaginase-etoposide (SMILE) chemotherapy regimen of asparaginase, dexamethasone, and methotrexate, which was briefly complicated by secondary hemophagocytic lymphohistiocytosis (HLH).

## Discussion

Sinusitis and facial swelling are common ED conditions, most frequently the result of an infectious etiology [1-2]. Despite the majority of these cases self-resolving with supportive care or with a brief course of antibiotics, the above case demonstrates the diagnostic and therapeutic emergency that NKTL presents. Extranodal NKTL arises from central necrosis within the nasal cavity, though it commonly presents after initial non-Hodgkin’s Lymphoma diagnosis or with rhinorrhea, nasal obstruction, and epistaxis [4-5]. It rarely presents with facial and periocular swelling, with only a few reported cases [5-7]. Literature has additionally reported NKTL concurrent with facial trauma and swelling [4]. Patients may present with regional lymphadenopathy in 15%-35% of cases [8-11]. The orbital extension is infrequent, highlighting the rare presentation of this deadly disease [10].

Patients rarely complain of fever, weight loss, or night sweats due to low cytokine secretion [4]. Similarly, the lack of laboratory inflammation is incongruent with the tissue destruction seen on imaging. No particular laboratory evaluation clinches this diagnosis, though elevated C-reactive protein, erythrocyte sedimentation rate, lymphocytosis or lymphopenia, and hyperglycemia may be seen [4, 8-9]. CT with contrast is the initial imaging modality of choice which can demonstrate tissue inflammation. However, MRI can better delineate bone destruction and neoplastic versus inflammatory processes, especially in the setting of superimposed bacterial sinusitis and orbital cellulitis [4]. Additionally, the patient’s exam demonstrated cranial nerve IX and X deficits; this finding reflects the leptomeningeal involvement, prompting expedited MRI to assess dural enhancement [12].

Any concern for superimposed bacterial or fungal sinusitis or orbital cellulitis should trigger appropriate antimicrobials, though definitive diagnosis requires tissue biopsy with histology [4]. Some reports suggest surgical management may be ineffective and lead to rapid disease progression; the mainstay of treatment is chemotherapy and radiation [10, 13]. Despite treatment, the prognosis for nasal NKTL remains poor with a five-year mortality rate of 10%-45% [4, 10]. Additionally, NKTL is a frequent cause of HLH; the development of this macrocyte hyperactivation immediately following or during treatment for NKTL further increases mortality up to 96.4% [14-15].

## Conclusions

Sinusitis is a common complaint in EDs usually of infectious etiology, but NKTL represents an uncommon but serious mimic. While the above patient demonstrated bacterial superinfection, she did not respond appropriately to antimicrobials or debridement, suggesting a more insidious underlying cause. This case illustrates the need for emergency clinicians to consider alternative diagnoses outside of infectious or allergic etiologies for atraumatic facial pre-septal edema. While facial lymphoma is a rare ED presentation, the majority of the literature focuses on follicular or B-cell lineage lymphoma, in contrast to this patient’s NKTL, highlighting the rare presentation of this uncommon ED diagnosis. Thus, subacute, asymmetric facial swelling should raise concern for potential malignancy when seen in combination with lymphopenia and symptoms refractory to antimicrobial therapy. Expeditious imaging and appropriate consultations may reduce the time to appropriate diagnosis and treatment, ultimately decreasing the morbidity and mortality of this rare presentation.
